# Age-related cardiac dysfunction is not associated with shorter telomere length in laboratory rats

**DOI:** 10.3389/fcvm.2026.1790767

**Published:** 2026-10-13

**Authors:** Sindre T. Strandberg, Trine B. Opstad, Andreas Romaine, Emil Espe, Mani Sadredini, Kari Gulbrandsen Løken, Sheryl Palmero, Jørgen A. Gravning, Ivar Sjaastad, Ida G. Lunde, Mathis K. Stokke, Ragnhild Helseth

**Affiliations:** 1 Oslo Center for Clinical Heart Research (Oslo-CCHR), Department of Cardiology Ullevaal, Oslo University Hospital, Oslo, Norway; 2 University of Oslo, Oslo, Norway; 3 Institute for Surgical Research, Oslo University Hospital, Oslo, Norway; 4 Institute for Experimental Medical Research (IEMR), University of Oslo and Oslo University Hospital, Oslo, Norway; 5 Department of Nursing and Health Promotion, Faculty of Health Sciences, Oslo Metropolitan University, Oslo, Norway; 6 KG Jebsen Center for Cardiac Biomarkers, Institute of Clinical Medicine, University of Oslo, Oslo, Norway

**Keywords:** age-related cardiac dysfunction, cardiac aging, cardiac telomere length, leukocyte telomere length, preclinical aging models, telomere length

## Abstract

**Introduction:**

Ageing is an independent risk factor for cardiovascular disease (CVD). Preclinical models of ageing are needed to better understand human diseases. Telomere shortening is a critical process in cellular ageing and senescence. In humans, it is well-established that telomere shortening in circulating leukocytes is associated with CVD, however, whether this applies to common preclinical models remains disputable. We investigated leukocyte telomere length (LTL), cardiac telomere length (CTL), and age-related cardiac remodeling and functional decline in commonly used laboratory rats.

**Methods:**

Sprague-Dawley rats (*n* = 51) were euthanized at 3, 9, 16, or 24 months (mo), respectively. Cardiac function was assessed by echocardiography. Relative telomere length, mRNA levels of telomerase and signature genes involved in inflammation and fibrosis were quantified in circulating leukocytes and left ventricular tissue by quantitative or digital droplet PCR (qPCR or ddPCR, respectively).

**Results:**

Rats with increasing chronological age (16/24 months compared to 9 months) exhibited cardiac hypertrophy and diastolic dysfunction, as evidenced by increased septal and posterior wall thickness, and elevated E/e’ ratio, and increased cardiac expression of central inflammatory (*Il6*) and fibrotic (*Ctgf*) genes. Relative LTL and CTL remained unchanged across chronological age groups (LTL: 9 months 1.000 vs. 16 months 0.939 vs. 24 months 1.120, *p* = 0.37; and CTL: 9 months 1.000 vs. 16 months 1.021 vs. 24 months 0.897, *p* = 0.42). CTL and LTL did not correlate (*r* = −0.027, *p* = 0.89). No association with cardiac dysfunction was observed, neither for LTL nor CTL.

**Discussion:**

Laboratory rats of high age exhibited cardiac hypertrophy and dysfunction, resembling human cardiac ageing, however we found no evidence of shorter telomere lengths in circulating leukocytes nor cardiac cells, suggesting a translational gap.

## 1 Introduction

Ageing is a well-known, independent risk factor for cardiovascular disease (CVD) (1). With the ageing global population, understanding the underlying mechanisms of age-related cardiovascular risk is crucial to meet the expected associated increase in CVD (2, 3). Telomeres are caps consisting of repetitive nucleotide noncoding tandem arrays (TTAGGG) at the terminal ends of all vertebrate chromosomes, that protect chromosomes from degradation and loss of genetic information during replication. Ageing is a complex process linked to cellular senescence, where telomere shortening is believed to be central (4). Accumulation of senescent cells and exhaustion of regenerative progenitors impair tissue repair and contribute to functional decline in multiple organs (5). Extensive studies in humans collectively show that leukocyte telomere length (LTL) in mid-life is associated with longevity (6). Shorter LTL is robustly linked to increased risk of CVDs like coronary artery disease (CAD) and heart failure (HF), with mendelian randomization studies supporting a causal relationship (7–9). Whole blood telomere length is associated with telomere length in several tissues (10), yet mechanistic links between LTL and organ-specific diseases are lacking (11). This is also true for the heart and myocardial disease. Preclinical research models are central to gain insight into mechanistic links between ageing and CVD, and rodents are the most frequently used. Here, we assessed relative LTL and cardiac telomere length (CTL) in ageing laboratory rats and evaluated their associations with age-related cardiac remodelling and dysfunction.

## 2 Methods

### 2.1 Ethical statement

The study was conducted at the Institute of Experimental Research (IEMR), Department of Comparative Medicine, and Oslo Center for Clinical Heart Research (Oslo-CCHR), Oslo University Hospital (OUH) Ullevaal. All procedures involving animals were approved by the Norwegian Food Safety Authority under FOTS ID 20208, and were carried out in compliance with national regulations for the care and use of laboratory animals and the ARRIVE 2.0 guidelines (12).

### 2.2 Animals

#### 2.2.1 Experimental design and animal details

Sprague-Dawley rats from ENVIGO were clinically healthy upon inclusion and had not been exposed to any experimental procedures. Rats that met predefined humane endpoints during the study were excluded (*n* = 12). A total of 51 male rats were included. Included animals were euthanized at one of four predefined age points: 3 months [*n* = 9, mean body weight (BW) 363 g], 9 months (*n* = 9, mean BW 506 g), 16 months (*n* = 13, mean BW 634 g), and 24 months (*n* = 20, mean BW 596 g). 9-months-old rats were considered adult and served as comparative controls for the other age groups, given that tibia length had stabilized (Table 1) and considering non-reproductive systems like brain development, immune system, bone growth, and social maturity (13).

**Table 1:** Age-related physiological and functional changes.

	3 months	9 months (control)	16 months	24 months	*p*
Biometric data	*n* = 9	*n* = 9	*n* = 13	*n* = 20	
Body weight (g)	360 (352, 376)	509 (497, 525)	646 (599, 662)***	577 (521, 662)**	**<0** **.** **01**
Tibia length (mm)	40.6 (40.2, 40.7)	43.7 (43.0, 44.8)	44.7 (44.3, 45.9)*	45.2 (44.1, 46.3)*	0.06
Heart weight (g)	1.1 ± 0.1	1.4 ± 0.1	1.6 ± 0.2*	1.8 ± 0.2***	**<0** **.** **01**
HW/TL^3^	16.1 (15.6, 17.0)	16.0 (15.0, 18.3)	17.6 (16.3, 18.6)	18.2 (17.1, 21.3)*	0.07
Lung weight (g)	1.4 ± 0.1	1.8 ± 0.1	2.1 ± 0.1**	2.2 ± 0.3***	**<0** **.** **01**
LW/TL^3^	22.0 (21.5, 22.5)	21.3 (19.3, 22.5)	22.7 (21.6, 23.9)	24.7 (20.3, 27.1)	**<0** **.** **05**
Echocardiography	*n* = 9	*n* = 9	*n* = 13^a^	*n* = 20^b^	
BPM	343 ± 24	323 ± 22	301 ± 19	300 ± 25*	**<0** **.** **05**
IVSd (mm)	1.53 ± 0.16	1.70 ± 0.15	2.15 ± 0.26***	2.23 ± 0.21***	**<0** **.** **01**
LVIDd (mm)	8.13 ± 0.32	9.26 ± 0.61	8.77 ± 0.54	8.97 ± 0.75	0.24
LVPWd (mm)	1.58 ± 0.14	1.68 ± 0.18	2.24 ± 0.23***	2.33 ± 0.19***	**<0** **.** **01**
LAD (mm)	4.18 ± 0.45	5.06 ± 0.54	5.38 ± 0.57	5.56 ± 1.00	0.32
FS (%)	36.1 (32.6, 36.9)	28.2 (23.8, 33.9)	30.2 (26.7, 33.3)	28.8 (21.0, 37.7)	0.96
LVEF (%)	63.6 (59.1, 64.8)	52.4 (45.6, 60.5)	55.3 (50.1, 59.8)	53.3 (40.8, 65.6)	0.98
e’	37.3 (36.2, 44.6)	37.9 (35.0, 39.0)	28.8 (23.5, 35.1)***	32.9 (28.0, 36.3)*	**<0** **.** **01**
E/e’	17.9 (16.8, 18.8)	16.7 (16.1, 18.9)	18.3 (17.1, 21.9)*	21.1 (17.6, 24.1)**	**<0** **.** **05**
Relative cardiac mRNA expression	*n* = 9	*n* = 9	*n* = 13	*n* = 19	
*Il6/Rpl4*	0.83 (0.71, 0.90)	1.00 (0.85, 1.18)	1.64 (1.37, 1.96)	2.44 (1.46, 5.20)**	**<0** **.** **01**
*Ctgf/Rpl4*	0.94 (0.83, 1.03)	1.00 (0.89, 1.15)	1.90 (1.75, 2.63)	4.38 (1.85, 5.71)***	**<0** **.** **01**
*Rlp4*	0.96 ± 0.08	1.00 ± 0.06	1.04 ± 0.09	0.97 ± 0.14	0.22

BPM, beats per minute, measured in M-mode; HW/TL^3^, heart weight/tibia length^3^; LW/TL^3^, lung weight/tibia length^3^; IVSd, interventricular septum diastolic diameter; LVIDd, left ventricular internal diastolic diameter; LVPWd, left ventricular posterior wall diastolic diameter; LAD, left atrial diameter; FS, fractional shortening; LVEF, left ventricular ejection fraction; *Il6*, interleukin 6; *Ctgf*, connective tissue growth factor; *Rpl4*, ribosomal protein L4*.* Values are given as mean ± SD or median (25, 75 percentiles). Statistical comparisons are performed by one-way ANOVA/Tukey's test or the Kruskal Wallis/Dunn's test as appropriate with the 9-month group as reference. The column *p* displays the *p*-value from either one-way ANOVA or Kruskal Wallis depending normal distribution or not.

a *n* = 11 for E’.

b *n* = 19 for E/e'.

* *p* < 0.05.

** *p* < 0.01.

*** *p* < 0.001.

Bold p-values refer to a significance level ≤0.05.

#### 2.2.2 Housing and husbandry

Rats were housed in open type III cages, located in racks and ventilated cabinets (Scantainers), where the incoming air was HEPA-filtrated. They lived in pairs, with access to standard chow and water *ad libitum*. Environmental conditions were controlled at 20 °C–24 °C, 45%–65% relative humidity, and a 12-h light/dark cycle. Enrichment included red polycarbonate tunnels and rat appropriate wood textures. All animals had been housed under stable conditions at the facility for an extended period prior to experimental procedures. Animals were monitored daily by trained personnel for signs of illness, distress, or injury, including changes in posture, coat condition, and activity.

### 2.3 Echocardiography

At the designated endpoints (3, 9, 16, and 24 months of age), animals underwent transthoracic echocardiography followed by blood sampling and tissue collection. Animals were anesthetized with isoflurane (induction: 3.5%, maintenance: 1.7% via face mask) and placed supine on a heated platform with ECG and respiratory monitoring. Transthoracic echocardiography was performed using a VEVO 3100 (VisualSonics, Toronto, Canada), software Vevo LAB (v.5.6.1, VisualSonics). The thoracic area was depilated with a depilatory cream to ensure optimal image quality. Parasternal long- and short-axis views were obtained. Total time under anesthesia was kept below 10 min per session. All measurements were analyzed offline by an investigator blinded to the age group, but not to animal ID or date of collection.

### 2.4 Blood sampling

Sampling of EDTA-full blood (*n* = 51) was performed at the endpoint under general anesthesia, separately from echocardiographic procedures. General anesthesia was induced with 4% isoflurane in 100% oxygen and maintained at 3% isoflurane via face mask. A midline abdominal incision was performed, and blood was collected from the inferior vena cava immediately prior to tissue harvest. Euthanasia was achieved by exsanguination. From *n* = 28 rats, blood was also collected into PAXGene tubes (Qiagen Nordic, Oslo, Norway) whereof unfortunately only *n* = 1 was from the 9-month-old group; the 16 months group was thus used for comparison in these analyses.

### 2.5 LV tissue

Following blood sampling, animals were maintained under deep isoflurane anesthesia as thoracotomy and excision of the heart from the great vessels (cardiectomy) was performed. The left ventricle (LV) of *n* = 51 rats was dissected free from surrounding tissue and symmetrically cut into two halves. The tissue was rinsed with at least three sequential dips in fresh, ice-cold PBS to remove any residual blood and gently blotted on clean paper. The tissue was then snap-frozen in liquid nitrogen and stored at −80 °C until further analysis. All tissue handling was completed within seven minutes of excision LV tissue was pulverized using pre-cooled equipment and maintained in liquid nitrogen.

### 2.6 Laboratory analyses

#### 2.6.1 Telomere length

Deoxyribonuclease (DNA) was extracted from LV tissue and circulating leukocytes by use of the PureLink Genomic DNA Mini Kit (Invitrogen by Thermo Fisher Scientific, CA, USA) and QiAamp DNA Blood Mini Kit (Qiagen GmbH, Hilden, Germany), respectively. Prior to DNA extraction, 25 mg of LV tissue was incubated at 55 °C for 3 h and 15’ in homogenized Genomic Digestion Buffer containing Proteinase K (Invitrogen), with vortexing at 400 rpm every 30'. DNA quality and quantity were measured on the NanoDrop, ND-100 (Saveen Werner, Sweden). Equal amount of DNA (1.0 ng/μL) was used to measure relative CTL and LTL with the Relative Rat Telomere Length Quantification qPCR Assay Kit (RRTLQ, cat #R8908 ScienCell Research Laboratories, CA, USA). This kit provides 2X GoldNStart TaqGreen qPCR master mix and sets of primers for telomere and the single copy reference (SCR). The telomere primer set recognizes and amplifies telomere sequences, while the single copy reference (SCR) primer set recognizes and amplifies a 100 bp-long region on rat chromosome 17, serving as reference, i.e., telomere length is reported as relative to this region. The 2X GoldNStart TaqGreen qPCR Master Mix is a SYBR Green dye-based qPCR master mix with a “hot-start” Taq DNA polymerase enzyme. PCR conditions for both targets were: an initial denaturation step (95° for 10 min) followed by 32 cycles of denaturation (95° for 20 s), annealing (52° for 20 s) and extension (72° for 45 s). A negative template control was included in each run to exclude contamination, and all samples were run in triplicates. All individual amplification curves for each sample were carefully validated and excluded if the standard deviation (SD) of the technical triplicates exceeded 0.5 Ct. Telomere lengths were successfully analyzed in *n* = 51 (all) and *n* = 34 (due to technical reasons) of the rat DNA samples from LV and circulating leukocytes, respectively.

#### 2.6.2 Gene expression analyses: LV tissue

Total RNA was extracted from tissue samples using the RNeasy Mini Kit (Qiagen, Cat. No. 74106, Qiagen Nordic, Oslo, Norway), and RNA concentration was measured with a MultiscanSky spectrophotometer (Thermo Fisher Scientific, Waltham, MA, USA). Complementary DNA (cDNA) was synthesized using the iScript cDNA Synthesis Kit (Bio-Rad Laboratories, Inc., Hercules, CA). mRNA levels of telomerase components (telomerase RNA component *Terc* (Rn03456487_s1) and telomerase reverse transcriptase *Tert* (Rn01409457_m1)) and central genes related to inflammation (*Il6* encoding interleukin 6 (Rn01410330_m1)) and fibrosis (*Ctgf* encoding connective tissue growth factor (Rn01537279_g1) were quantified using droplet digital PCR (ddPCR), with *Rpl4* (Rn00821091_g1)) serving as housekeeping gene. ddPCR was performed on a QX200 (Bio-Rad Laboratories, Inc., Hercules, CA), and analysis was conducted using QuantaSoft (Bio-Rad). *Rpl4* was run on a separate plate. We omitted one 24-month old rat from the LV gene expression analyses of *Il6* and *Ctgf* due to a malignant, non-cardiac tumor that could have affected systemic inflammation.

#### 2.6.3 Gene expression analyses: circulating leukocytes

Total RNA was extracted from circulating leukocytes using the PAXGene Blood RNA kit (Qiagen GmbH, Hilden, Germany). The PAXGene tubes were initially adapted to available blood in rats using 1 mL of blood to 4.14 mL of stabilizing RNA reagents. RNA quality and quantity were quantified on the NanoDrop, ND-100 (Saveen Werner, Sweden). RNA samples were diluted with RNase-free water to a final concentration of 5 ng/μL. cDNA was synthesized using the qScriptTM cDNA SuperMix (Quanta Biosciences, VWR). Quantitative real-time PCR (qPCR) was performed on the VIIa7 instrument using the TaqMan Universal PCR Master Mix, No AmpErase UNG, and the following TaqMan assays: *Tert* (Rn01409457_m1), *Terc* (Rn03456487_s1), and normalized to the housekeeping gene *Rpl4* (Rn00821091_g1) (all Applied Biosystems). PCR conditions were: an initial denaturation step at 95 °C for 10 min followed by 40 cycles of denaturation at 95 °C for 15 s, annealing and extension at 60 °C for 60 s. A negative template control was included in each run to exclude contamination, and all samples were run once. Expression levels were successfully analyzed in *n* = 27 of the 28 samples, except for the *Terc* analysis (*n* = 26) where one sample was omitted for technical reasons.

### 2.7 Statistical analyses

The distribution of the data was evaluated by histograms, QQ plots and the Shapiro–Wilk test. For normally distributed variables, data are given as mean (SD), differences across age groups evaluated by One-Way ANOVA and Tukey's test, and difference between two groups assesses by Student's *t*-test. For non-normal variables, data are given as median (25, 75 percentiles), differences across age groups evaluated by the Kruskal Wallis and Dunn's test, and difference between two groups assessed by the Mann–Whitney test. To investigate the relationship between telomere length and multiple markers of cardiac dysfunction, we employed multivariate multiple regression analysis. This statistical approach was selected to account for any interdependence of the cardiac parameters as they probably reflect overlapping aspects of cardiac dysfunction. Non-normal parameters were log-transformed before included in the model. Correlation analyses were performed by Spearman's Rho. The level of statistical significance was set to *p* < 0.05 (two-sided). All statistical analyses were performed in R version 4.4.1 in RStudio and STATA/SE 18.0 (Statacorp).

## 3 Results

### 3.1 Age-related cardiac remodelling

We investigated male Sprague-Dawley laboratory rats over 2 years of laboratory ageing. Chronological age was associated with cardiac hypertrophy as measured by echocardiography. The diastolic diameter of the interventricular septum and the posterior wall both increased from 9 to 16 and 24 months, respectively (Table 1). Chronological age was also associated with increased diastolic filling pressures as measured by peak early mitral inflow velocity (E) divided by the early diastolic mitral annular velocity (e’): The E/e’ increased significantly from 9 to 16 and 24 months, respectively (Table 1). Accordingly, the cardiac tissue showed increased expression of signature genes involved in inflammation (*Il6*) and fibrosis (*Ctgf*) (Table 1).

### 3.2 Cardiac telomere length (CTL)

#### 3.2.1 In ageing

CTL did not change with chronological age (Figure 1a). Cardiac expression of the telomerase enzyme component *Tert* was higher at 16 months, while the RNA template part of the telomerase enzyme, *Terc*, was stable across age groups (Figures 1b,c).

**Figure 1:**
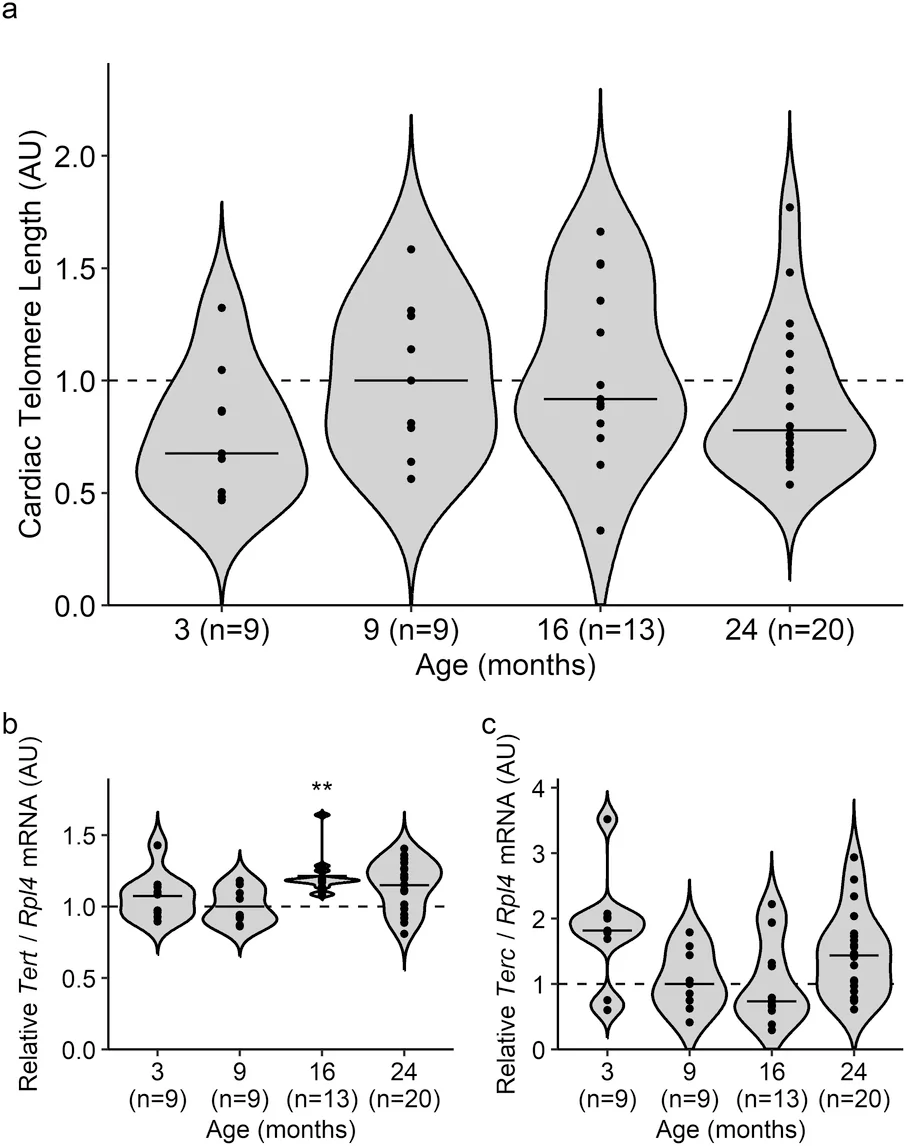
Cardiac telomere length (CTL) and left ventriclular (LV) gene expression across age. **(a)** Cardiac telomere length, **(b)** *Tert*, and **(c)** *Terc*. Violin plots show individual data points and group median **(a,c)** or mean **(b)**, based on data distribution; the dashed line indicates the 9-month reference value used for statistical comparisons. Differences across age groups were evaluated by the Kruskal–Wallis test/Dunn's test **(a,c)** or by one-Way ANOVA and Tukey's test **(b)**. AU, arbitrary units. **p < 0.01.

To explore whether an “ageing” phenotype at the tissue level was associated with CTL, rats were stratified into high and low *Il6* and *Ctgf* expression. The threshold was set to the mean value for 9-month old rats + >2 SDs. A total of 13 rats had high *Il6,* while 18 rats had high *Ctgf*. The CTL did not differ between rats with high and low *Il6*, nor between rats with high and low *Ctgf* (Figure 2).

**Figure 2:**
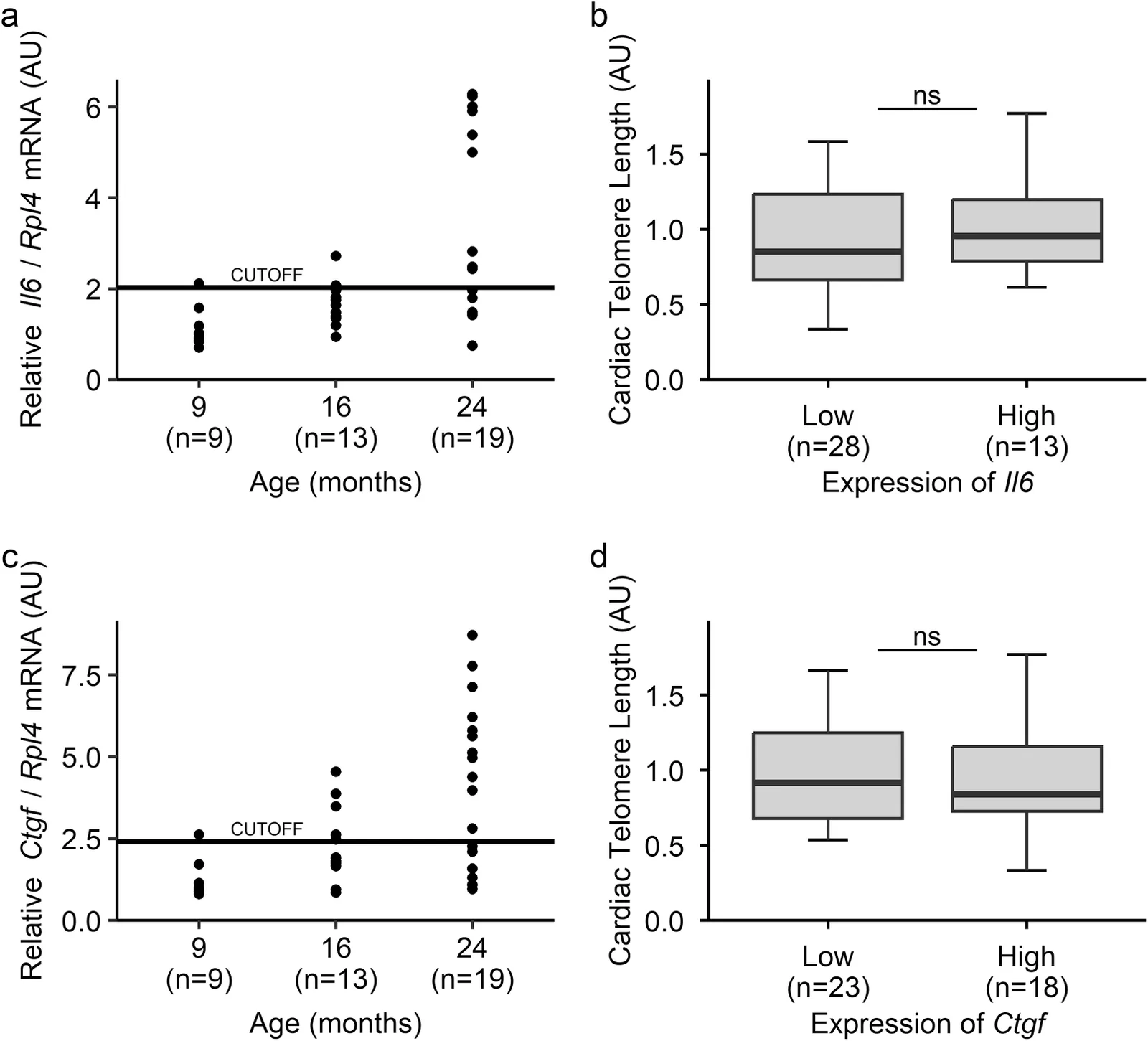
Cardiac telomere length (CTL) stratified by advanced ageing. **(a)** *Il6/Rpl4* across age groups. **(b)** Cardiac telomere length in rats with low vs. high cardiac *Il6* levels. **(c)** *Ctgf/Rpl4* across age groups. **(d)** Cardiac telomere length in rats with low vs. high cardiac *Ctgf* levels. Cutoff for low vs. high expression was set at the mean + >2 SD of the 9-month group. Statistical test Mann–Whitney *U*-test. Box plots express median (25, 75 percentiles) and 1.5*interquartile range (whiskers). *Il6*: interleukin 6. *Ctgf*: connective tissue growth factor. *Rpl4:* Ribosomal Protein L4. AU, arbitrary units; ns, not significant. One 24-month-old rat was excluded due to a malignant tumor.

#### 3.2.2 In cardiac dysfunction

We stratified rats based on signs of cardiac dysfunction defined by cut-off values shown in Figure 3. The cutoff was set to the mean value + >2 SDs at 9-months, except from LVEF where the threshold was set to 40% to reflect the traditional clinical definition of HF with reduced ejection fraction (HFrEF). A total of 7 rats had high heart weight; 11 had high lung weight; 9 had large left atrial diameter; 9 had low LVEF; and 13 had high E/e'. Only high heart weight was associated with shorter CTL (Figure 3). Tested collectively, the markers of cardiac dysfunction were not associated with the CTL (Table 2).

**Figure 3:**
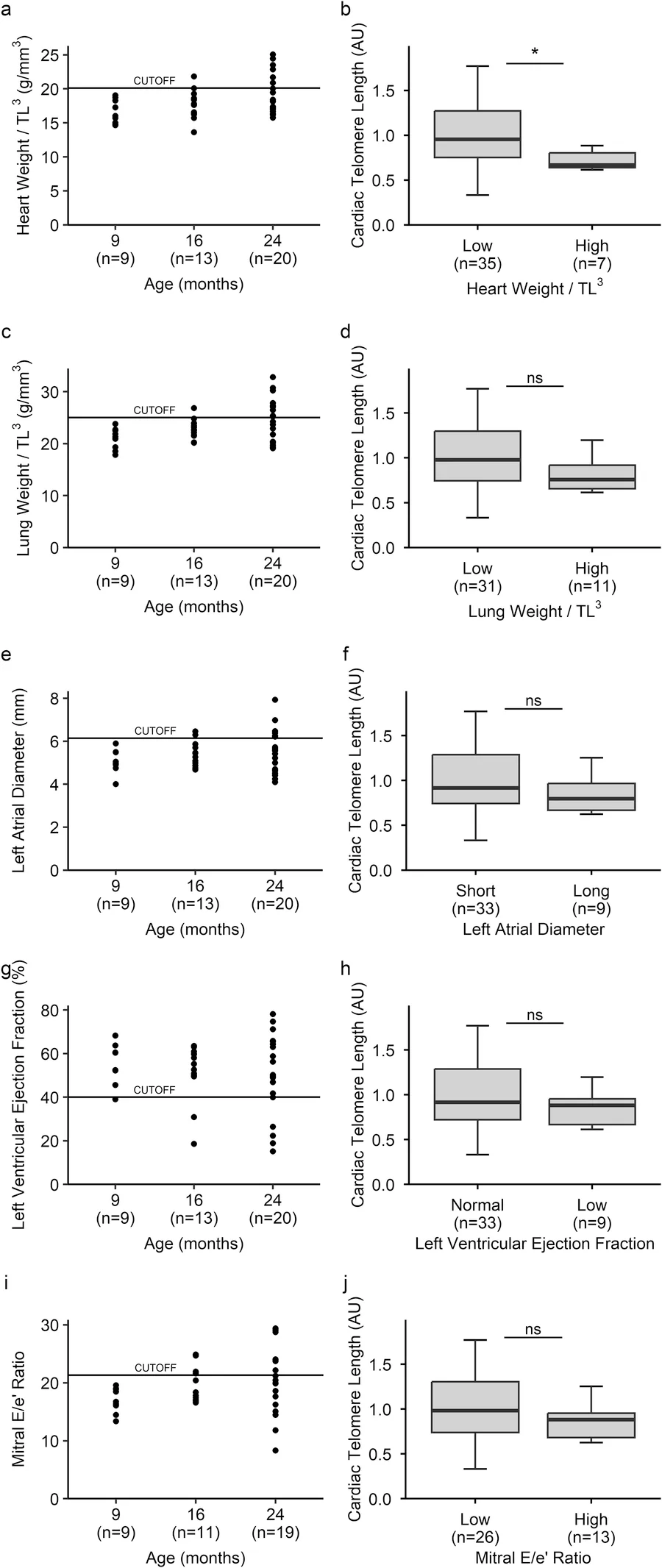
Cardiac telomere length (CTL) stratified by signs of cardiac dysfunction. **(a)** Heart Weight/TL^3^ across age groups. **(b)** Cardiac telomere length in rats with low vs. high Heart Weight/TL^3^. **(c)** Lung Weight/TL^3^ across age groups. **(d)** Cardiac telomere length in rats with low vs. high Lung Weight/TL^3^. **(e)** Left Atrial Diameter across age groups. **(f)** Cardiac telomere length in rats with short vs. long Left Atrial Diameter. **(g)** Left Ventricular Ejection Fraction across age groups. **(h)** Cardiac telomere length in rats with normal vs. low LVEF. **(i)** Mitral E/e’ Ratio across age groups. **(j)** Cardiac telomere length in rats with low vs. high Mitral E/e’ Ratio. Cutoff for stratification was set at the mean + >2 SD of the 9-month group, except for LVEF, where the cutoff was set at 40% to reflect the traditional clinical definition of heart failure with reduced ejection fraction (HFrEF). Statistical test Mann–Whitney *U* test. Box plots express median (25, 75 percentiles) and 1.5*interquartile range (whiskers). TL, Tibia length; AU, arbitrary units; ns, not significant, * *p* < 0.05.

**Table 2:** Cardiac telomere length and signs of cardiac dysfunction.

Echocardiographic markers	Beta	SE	95% CI	*p*	*R* ^2^
Log HW/TL^3^	−0.088	0.066	−0.22 to 0.05	0.19	0.046
Log LW/TL^3^	−0.049	0.067	−0.19 to 0.09	0.47	0.014
LAD	−0.396	0.383	−1.17 to 0.38	0.31	0.028
Log E/e’	−0.089	0.102	−0.30 to 0.12	0.39	0.020

Multivariate multiple regression analysis. SE, standard error; CI, confidence interval; HW/TL^3^, heart weight/tibia length^3^; LW/TL^3^, lung weight/tibia length^3^; LAD, left atrial diameter.

### 3.3 Leukocyte telomere length (LTL)

#### 3.3.1 In ageing and cardiac dysfunction

LTL did not differ between chronological age groups, neither did the leukocyte expression of *Tert* and *Terc* (Figures 4a–c). Comparisons for *Tert* and *Terc* were only made between 16- and 24-month-old rats, as only one rat (*n* = 1) had available leukocyte mRNA samples at 9 months of age. High cardiac *Il6* mRNA levels were not associated with LTL (1.02 ± 0.19 vs. 1.02 ± 0.34, *p* = 0.96), neither was high cardiac *Ctgf* (0.92 ± 0.24 vs. 1.02 ± 0.32, *p* = 0.14). LTL was not associated with markers of cardiac dysfunction (Table 3).

**Figure 4:**
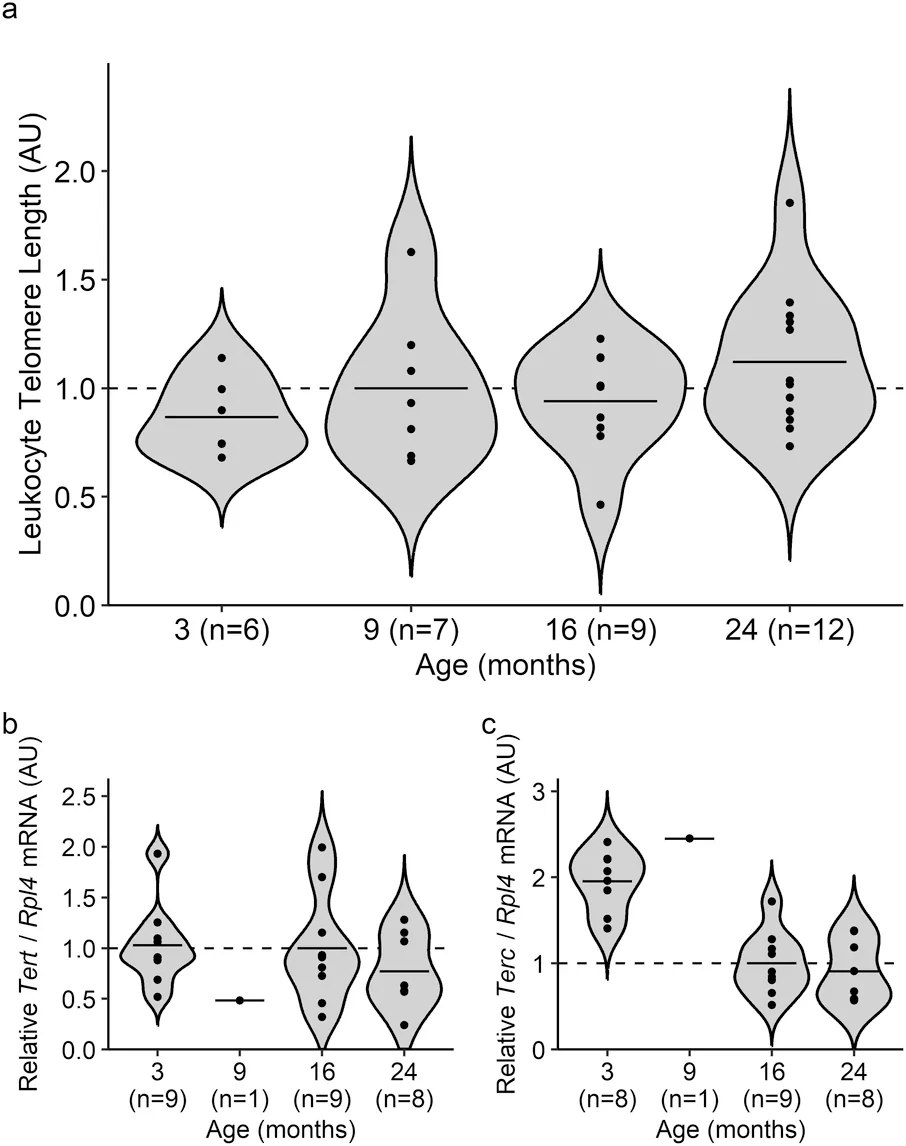
Leukocyte teleomere length (LTL) and telomerase gene expression across age. **(a)** Leukocyte telomere length, **(b)** *Tert* **(c)** *Terc*. Violin plots show individual data points and group mean; the dashed line indicates the reference value used for statistical comparisons: 9-month **(a)** or 16-month **(b,c)**. Differences across age groups evaluated by One-Way ANOVA and Tukey's test. AU, arbitrary units; *Rpl4*, Ribosomal Protein L4.

**Table 3:** Leukocyte telomere length (LTL) and signs of cardiac dysfunction.

Echocardiographic markers	Low		High		*p*
HW/TL^3^	1.02 ± 0.31		1.09 ± 0.25		0.70
LW/TL^3^	1.02 ± 0.32		1.06 ± 0.24		0.80
LAD (mm)	1.02 ± 0.35		1.05 ± 0.21		0.82
LVEF (%)	1.01 ± 0.25		1.04 ± 0.32		0.84
E/e’	1.03 ± 0.34		1.08 ± 0.21		0.74
Multivariate regression analysis	Beta	SE	95% CI	*p*	*R* ^2^
Log HW/TL^3^	0.082	0.960	−0.11 to 0.280	0.40	0.029
Log LW/TL^3^	−0.005	0.106	−0.22 to 0.21	0.96	0.000
LAD	−0.771	0.589	−1.99 to 0.45	0.20	0.067
Log E/e’	0.121	0.130	−0.15 to 0.39	0.36	0.035

Student *t* test and multivariate multiple regression analysis. SE, standard error; CI, confidence interval; HW/TL^3^, heart weight/tibia length^3^; LW/TL^3^, lung weight/tibia length^3^; LAD, left atrial diameter; LVEF, left ventricular ejection fraction. Values are given as mean ± SD.

### 3.4 Telomere length in cardiac tissue vs. circulating leukocytes

LTL and CTL were not correlated (Figure 5).

**Figure 5:**
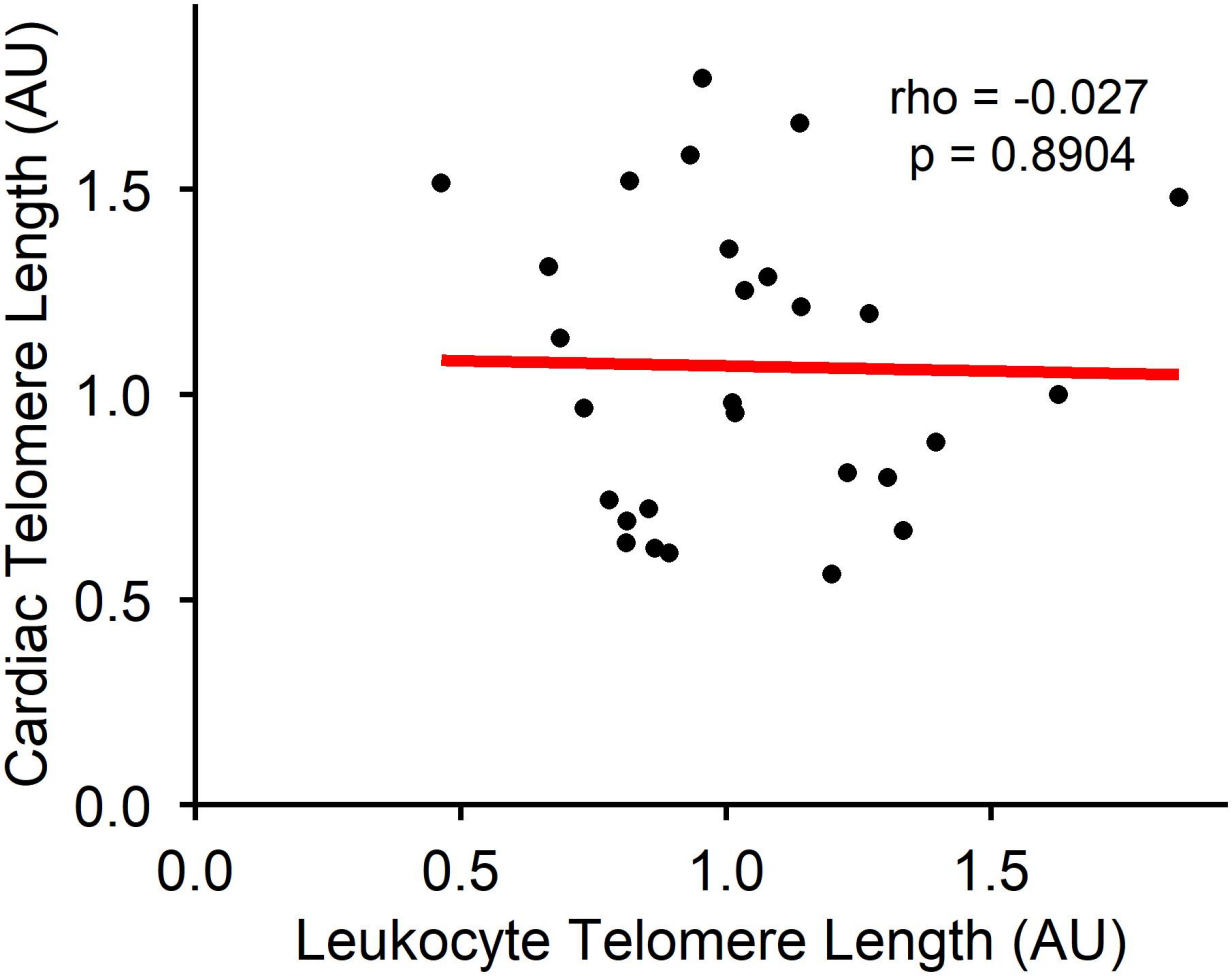
Correlation between leukocyte and cardiac teleomere lengths (LTL and CTL). Statistical test Spearmans rho. AU, arbitrary units.

## 4 Discussion

Sprague-Dawley rats allowed to age over 2 years in the laboratory showed a cardiac phenotype that mimicked changes seen in humans with increasing chronological age, i.e., hypertrophy, high filling pressures, inflammation and fibrosis. Over this considerabley long time-span, we found no evidence of shorter relative telomere lengths in circulating leukocytes nor cardiac cells. Moreover, we found no associations between measures of age-related cardiac dysfunction and relative telomere length, neither CTL nor LTL. This adds information to preclinical research on cardiovascular ageing and disease, and suggests that ageing Sprague-Dawley laboratory rats should be used with caution when investigating telomere length-related ageing processes.

In humans, LTL shortens with age and is associated with increased mortality and CVD (7, 14, 15). One meta-analysis of 24 studies and almost 44.000 participants found that short LTL was an independent risk factor for CAD (7). Telomere shortening in leukocytes is thought to reflect the complex process of biological ageing, and the association to CVD is assumed to involve cellular senescence and inflammation (16). Mendelian randomization analyses have linked LTL to HF in humans (8, 17, 18). These associations might be especially relevant for HF with preserved ejection fraction (HFpEF), associated with age and inflammation (19).

Compared to LTL, data on the association between ageing and CTL is less clear and the sparsity of human LV tissue available to research is one of the main resons why preclinical models are so instrumental in CVD research. Several studies do suggest that CTL does not shorten with advancing chronological age in humans (20–22). At the same time, patients with HF have short CTLs which coincides with increased cardiac apoptosis (20, 23, 24). Our rats had cardiac dysfunction, without signs of overt HF. The 24-month life span is a relevant time frame to explore cardiac dysfunction as about 1 out of 4 (26%) of the 24-month old rats in our study had LVEF < 40%. Further, we used LV tissue, which contains both cardiomyocytes and non-cardiomyocyte cells (25). Our neutral CTL results thus reflects the total cellular content of the myocardium, not only cardiomyocytes, and could also be attributed to the relatively non-mitotic nature of cardiomyocytes (26). Our observations are, however, in line with previous reports where only a small fraction of myocytes from old rats displayed telomeric shortening (27). Comparison of telomere lengths from numerous tissues are needed to settle how telomere dynamics change with age and in relation to mitotic capacities.

Our study suggests that LTL is not associated with chronological age and age-related cardiac dysfunction in Sprague-Dawley laboratory rats. In humans, shorter LTL is robustly linked to increased risk of CAD and HF, with mendelian randomization studies supporting a causal relationship (7–9). Thus, our results point to a discrepancy between human and rat biology and pathobiology, thus suggesting a critical translation gap, given that rats are key preclinical models for CVD.

Although it is well-established that telomere shortening in circulating leukocytes is associated with CVD in humans, whether this applies to common preclinical models such as rats remains disputable. Our results add further evidence to this field of research. In contrast to our findings, previous rodent studies have indeed linked LTL to age and cardiac dysfunction, and shown that telomerase knock-out mice (Terc^−/−^) have reduced cardiomyocyte proliferation, increased cell death, cellular hypertrophy, and impaired cardiac function (28–30). However, a recent study of rodents found no difference in relative LTL when comparing aged to young leukocytes (11), in line with our results. Furthermore, an age-related shortening of relative LTL was only observed in a small subset of the tested tissues. Our results are also in line with other rodent studies where telomere-related variables do not change with increasing age (31). These various findings could be attributable to differences in rodent species and lines, but could also stem from methodological aspects of measuring telomere lengths.

Multiple molecular methods have been developed to measure telomere length, suited for different purposes, number of samples, and DNA quantity and quality. These methods are not directly comparable, they vary reproducibility, and they all carry advantages and disadvantages (32, 33). Terminal restriction fragment (TRF) analysis is the original technique that has been used in numerous publications (34), measuring mean, i.e., non-chromosome-specific, absolute length of genomic telomeres through restriction digestion. It requires large amounts of DNA and is labor intensive, and not suited for large populations. Here, we have measured mean LTL and CTL relative to the SCR using qPCR, developed by Cawthon in 2002, which is a frequently used method suited for large sample sizes and with relatively little DNA available, e.g., blood samples from humans (35). Absolute telomere length can also be measured using qPCR, i.e., aTL qPCR, developed in 2011 by O'Callaghan and Fenech, using a standard curve of known telomere lengths. aTL qPCR measures the exact length of telomeres expressed in kb per diploid genome (36). Such qPCR methods carry relatively low cost and are high-throughput, however they are not readily comparable between studies. Single telomere length analysis (STELA) is qPCR-based, aimed at measuring individual chromosome termini, which is especially important since single critically short telomere lengths are suggested as lead signals to cellular senescence. Fluorescence *in situ* hybridization (FISH) techniques are chromosome-specific with the input analyte being chromosomes or nuclei rather than DNA. Classical FISH methods hace limited throughput, although newer variants including high-throughput quantitative FISH (HT Q-FISH) and array-based FISH, allow for higher sample volumes (37). Nowadays, next-generation sequencing (NGS) use short and long reads to infer telomere content and length distribution. Thus, our main finding of no association between relative LTL nor CTL with chronological age or cardiac dysfunction in ageing laboratory rats needs to be interpreted with the underlying notion that it was measured by the relative telomere length qPCR method.

### 4.1 Limitations

The limitations of our study comprise lack of serial blood samples, which would have provided age-related changes in individual rats. Only male rats were included, limiting the research's generalizability to both sexes. Our study would have been strengthened by parallell measurement of absolute telomere length, e.g., aTL qPCR, or NGS quantification.

### 4.2 Conclusion

In the preclinical Sprague-Dawley rat model, which displayed a cardiac phenotype resembling human ageing over 2 years, we found no changes in neither leukocyte nor cardiac telomere lengths, suggesting a translational gap vs. humans, where shortening telomere lengths are well-established in ageing.

## Data Availability

The raw data supporting the conclusions of this article will be made available by the authors, without undue reservation upon reasonable request.
